# Medication Error Management in Private Hospitals in Costa Rica: A Mixed-Methods Evaluation of Practices and Improvement Strategies

**DOI:** 10.7759/cureus.78693

**Published:** 2025-02-07

**Authors:** Jorge Arturo Villalobos-Madriz, Esteban Zavaleta-Monestel, Ernesto Martínez-Vargas, Abigail Fallas-Mora, Melissa Prado-Rivero, Natalia Bastos-Soto, Gary Cochran

**Affiliations:** 1 Faculty of Pharmacy, Universidad Latina de Costa Rica, San José, CRI; 2 Faculty of Pharmacy, Hospital Clinica Biblica, San José, CRI; 3 Faculty of Pharmacy, Hospital Clinica Biblica, San Jose, CRI; 4 Faculty of Medicine, University of Nebraska Medical Center, Omaha, USA

**Keywords:** hospital pharmacy, medication discrepancies, medication errors, patient safety, risk management

## Abstract

Introduction: Medication errors pose significant risks to patient safety and healthcare quality. This study analyzes the practices of identification and management of these errors in private hospitals in Costa Rica from January 2020 to January 2024.

Methodology: A cross-sectional survey was conducted in four private hospitals, evaluating 167 items related to 11 key drug management processes. In addition, a detailed analysis of retrospective data was carried out at one of the participating hospitals to delve deeper into the nature of the errors.

Results: The hospitals presented an overall level of implementation of 74% in the practices evaluated. The prescription and transcription stages were the most vulnerable, while most errors were detected before affecting patients. The drugs involved were mainly of general use and high risk.

Conclusion: The results highlight the importance of using tools based on international standards adapted to the local context. These help identify areas for improvement, promote preventive strategies, and strengthen patient safety. Applying this approach in other settings could drive standardization and improve outcomes in medication management.

## Introduction

Medications are the most widely used medical intervention worldwide. However, the increase in medication use goes hand in hand with an increased occurrence of medication errors [1]. These represent one of the leading causes of preventable injuries within healthcare systems around the world [2].

A medication error refers to any preventable incident caused by the improper use of medications, which can lead to harm to the patient [1]. They can occur at any stage of the medication process, including manufacturing, packaging, storage, and distribution, all the way to prescribing, transcription, review, preparation, labeling, dispensing, administration, and follow-up [1, 3]. These errors are related to professional practice, products, procedures, and healthcare systems [1].

It is estimated that one in 10 patients suffers some harm in a healthcare setting, and one in 20 can be attributed to a medication error, of which half are preventable [4]. In the United States, it represents the sixth most common cause of death, accounting for injuries to approximately 1.3 million people in a one-year period [5,6]. According to data from the World Health Organization, it is estimated that annually, medication errors represent an expense of 42 billion dollars per year for the health sector [4].

The private healthcare system in Costa Rica complements the robust public healthcare system, offering high-quality services with a focus on advanced technology, personalized care, and shorter wait times. Internationally, it stands out for its growing adoption of global standards of quality and safety in care, including the management of medication errors. However, compared to systems in developed countries, challenges remain related to the homogeneous implementation of safe practices and equitable access to technological and training resources. These factors reflect a developing context, where the integration of assessment tools and preventive strategies, adapted to local needs, is key to closing gaps in patient safety [7-10].

Given this, the objective of this work is to analyze the processes of identification and management of medication errors in private hospitals in Costa Rica during the period of January 2020 to January 2024 through the application of a survey to these hospitals. Through this analysis, we seek to describe the nature and frequency of medication errors that occur in these settings, develop an evaluation tool based on international guidelines to measure the effectiveness of medication error management systems, and evaluate the level of implementation of preventive and corrective strategies in the selected hospitals.

The study also aims to identify critical areas for improvement in drug management and provide specific recommendations that allow hospital institutions to optimize the safety of their pharmacotherapeutic processes, with the aim of protecting patients' health and reducing the incidence of adverse drug-related events.

## Materials and methods

This study employed a mixed-methods design, integrating a cross-sectional survey with a retrospective analysis of medication error data. This approach was selected to provide a comprehensive understanding of medication error identification and management systems in private hospitals in Costa Rica from January 2020 to January 2024. This study was conducted at Hospital Clinica Biblica, San José, Costa Rica.

The mixed-methods design leveraged the survey to evaluate the implementation of safety practices, identifying strengths and areas for improvement across multiple key processes. The retrospective analysis, in turn, provided detailed insights into the most vulnerable stages, types of errors, and medications involved in a specific hospital. This combination of quantitative and qualitative data ensured a holistic perspective, enhancing the findings' validity and practical applicability.

Sampling method

The study employed purposive sampling to select hospitals and participants, aiming to capture data from a diverse range of healthcare settings. Four Costa Rican private hospitals with varying levels of complexity were included: Hospital A, Hospital B, Hospital C, and Hospital D. Inclusion criteria required hospitals to have established medication management processes and available data on medication errors during the study period. Hospitals were excluded from the study if they lacked complete or consistent records on medication errors during the analysis period, did not have formal protocols for medication management, did not grant authorization to participate, or were not operational throughout the entire study period.

Hospital A has 75 beds, five specialized services, 20 operating rooms, 18 specialties, and three accreditations. Hospital B, with 10 beds, offers three specialized services, two operating rooms, and 28 specialties and has no accreditations. Hospital C features 11 beds, five specialized services, five operating rooms, and 36 specialties and also lacks accreditations. Finally, Hospital D, the largest among the group, has 234 beds, five specialized services, eight operating rooms, 25 specialties, and seven accreditations.

Although an evaluation instrument was applied to four private hospitals in Costa Rica, the specific information related to medication errors and their classification was obtained exclusively from Hospital A. This is because this hospital was the only one with complete data on the occurrence of errors across the various stages of the medication process during the study period. Therefore, the detailed analysis of medication errors is limited solely to this institution.

Evaluation tool development and validation

The evaluation tool was developed based on international standards, incorporating guidelines from the American Society of Health-System Pharmacists (ASHP), Joint Commission International (JCI), and the European Medicines Agency (EMA). Designed to comprehensively assess medication management practices, the tool included 167 items organized into 11 key processes. These processes were: planning of safe medication practices, with seven items; organization and management, with 11 items; selection and acquisition of medicines, comprising 23 items; storage, with 29 items; patient admission, covered by one item; medical orders, transcription, and review, with 27 items; preparation and dispensing, including 12 items; administration, with 16 items; follow-up, covered by 18 items; patient discharge, with two items; and evaluation, addressed by one item.

The tool was reviewed and validated by an interdisciplinary committee comprising hospital pharmacists, patient safety specialists, and physicians. The committee ensured that the items comprehensively addressed vulnerabilities at each critical stage of the medication management process.

Data collection

The survey was conducted in January 2024 and was administered in Spanish to professionals responsible for medication error data in each hospital. For publication purposes, the responses were translated into English. Participants evaluated the level of implementation for each item using a three-tiered scale: fully implemented, indicating total compliance; partially implemented, reflecting limited or deficient compliance; and not implemented, signifying no evidence of compliance.

Data analysis

Quantitative data from the survey were analyzed using descriptive statistics to identify patterns, strengths, and areas for improvement. The retrospective analysis of medication error data involved categorizing errors by stage, type, and medication involved. Results from both components were integrated to identify common vulnerabilities and inform tailored strategies for risk mitigation.

## Results

Medication error management process assessment tool

A total of 167 items were evaluated and distributed across 11 key processes related to medication error management. The details of these processes and their corresponding evaluations can be found in Appendix A. Table 1 summarizes the individual performance of each hospital as evaluated by the tool.

**Table 1:** Hospital-wise results for the evaluation of medication errors Note: This study is descriptive in nature without direct statistical comparisons due to structural differences between the hospitals.

	Implemented (%)	Partially implemented (%)	Not implemented (%)
Hospital A	80	15	5
Hospital B	60	30	10
Hospital C	74	20	6
Hospital D	63	27	10

**Figure 1:**
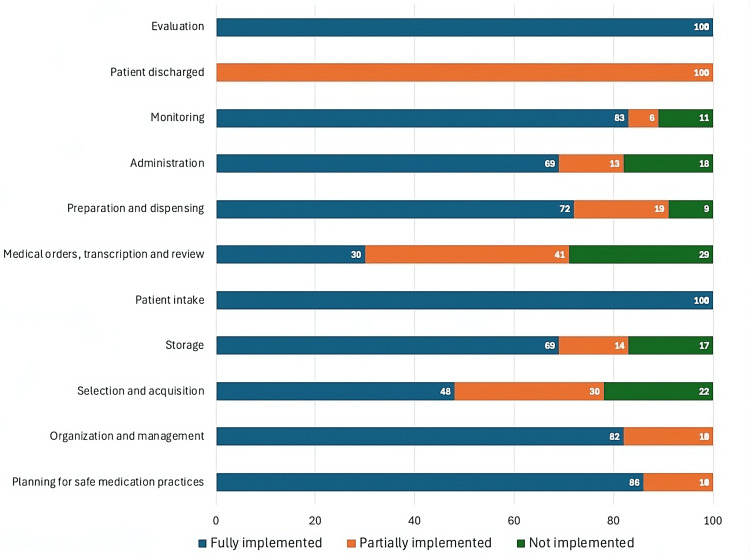
Level of implementation of the processes evaluated by the Medication Error Management Process Assessment Tool in Hospital A

**Figure 2:**
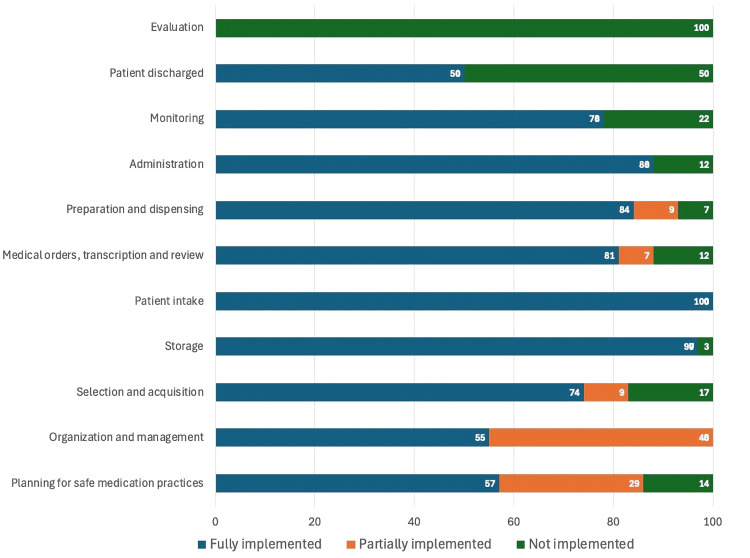
Level of implementation of the processes evaluated by the Medication Error Management Process Evaluation Tool in Hospital B

**Figure 3:**
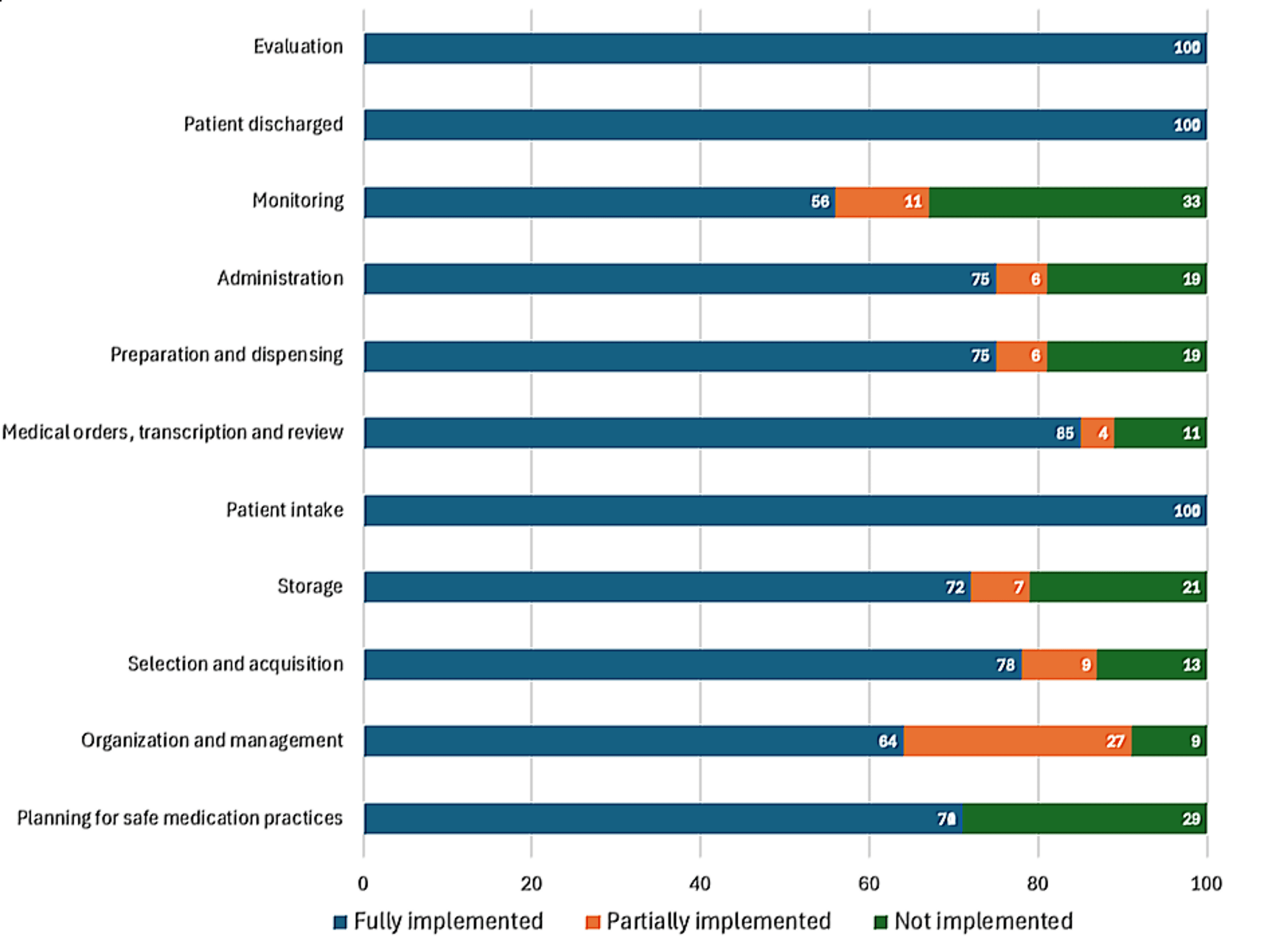
Level of implementation of the processes evaluated by the Medication Error Management Process Evaluation Tool in Hospital C

**Figure 4:**
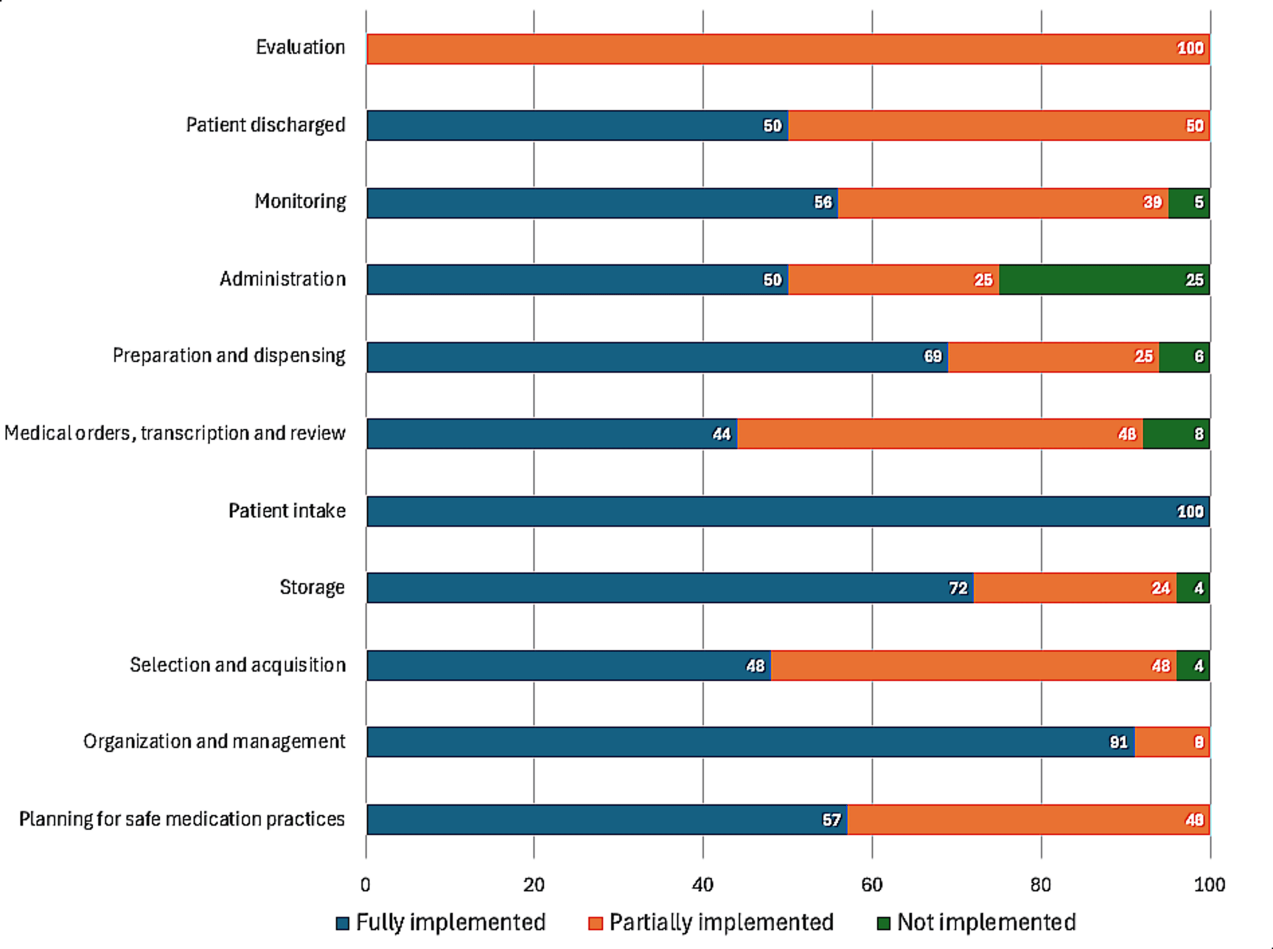
Level of implementation of the processes evaluated by the Medication Error Management Process Evaluation Tool in Hospital D

Figures 1-4 represent the performance of each of the four hospitals in each of the 11 key processes assessed by the Medication Error Management Process Assessment Tool. Of these, it is Hospital A had the highest percentage of processes fully implemented, and Hospitals B and D had the highest percentage of processes classified as "not implemented".

Medication errors

The specific data on medication errors analyzed in this study come only from Hospital A. Although the assessment instrument was applied in four hospitals, Hospital A was the only one that provided complete information on the occurrence of errors in the different stages of the medication process. For this reason, the results classified in terms of stages of the process, severity categories (The National Coordinating Council for Medication Error Reporting and Prevention (NCC MERP)), and types of drugs involved are limited to this institution, allowing for an in-depth analysis of their situation.

**Figure 5:**
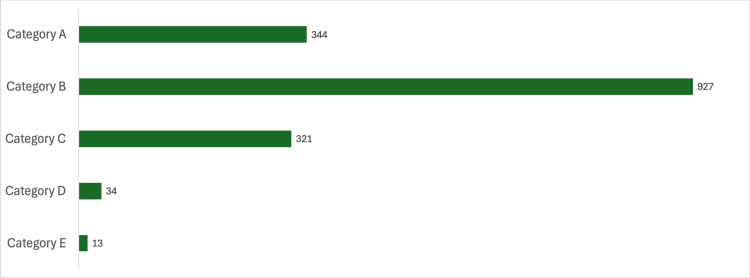
Classification of medication errors according to their severity as per the NCC MERP classification NCC MERP: The National Coordinating Council for Medication Error Reporting and Prevention

**Figure 6:**
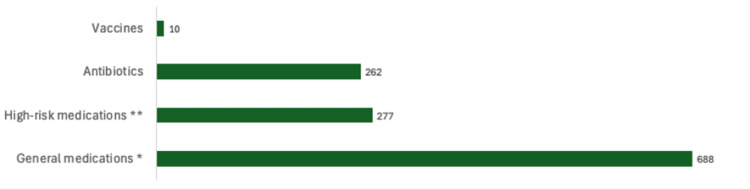
Classification of medication errors according to medication categories * Drugs of wide applicability used in clinical practice for the management of common or less critical diseases; **Medications that carry an increased risk of causing significant harm to the patient when used incorrectly (e.g., blood thinners, anti-cancer agents, concentrated electrolytes).

During the period between January 2020 and January 2024, a total of 1,605 medication errors were committed in Hospital A, which were classified according to the stage of the medication process where the error was made (Table 2), according to severity (Figure 5), and according to the type of medication (Figure 6).

**Table 2:** Classification of medication errors by stages Note: This study is descriptive in nature without direct statistical comparisons due to structural differences between the hospitals.

Stage of the process at which the error was made	Number of errors made, n(%)
Prescription	706 (43.07)
Transcription	494 (30.14)
Dispensation	132 (8.05)
Labeling	125 (7.63)
Administration	111 (6.77)
Storage	41 (2.50)
Conciliation	29 (1.77)
Monitoring	1 (0.07)
Total	1639 (100)

Figure 5 shows the classification of errors according to their severity according to the classification of the NCC MERP. It is important to note that most of the errors occurred in Category B and that no errors were reported in Categories F, G, H, or I.

## Discussion

Medication errors pose a serious threat to patient safety. The main objective of drug therapy is to improve the health and quality of life of those who use it. However, in any healthcare system, errors can occur at various stages of the process, from the storage of medications to their prescription, dispensing, administration, and use by the patient [2,11,12]. These errors have the potential to compromise both the effectiveness of the treatment and the health of the patient, and many of them are perfectly avoidable [11,13].

Improper use of medications can cause harm to the patient when a safe and rational medication use program is not used. Given this, it is of utmost importance that medical staff work together with pharmacists and nurses to provide timely follow-up of pharmacotherapy, especially when reports show that pharmaceutical clinical involvement helps reduce the rate of preventable adverse effects by medications by 78% [14,15].

The evaluation tool developed in this study identified strengths and critical areas for improvement in medication error management systems in Costa Rican private hospitals. Being based on international standards such as those of the ASHP, JCI, and EMA and validated by interdisciplinary professionals in hospital pharmacy, it allows not only a comprehensive analysis of key processes in medication management but also an objective comparison with globally accepted practices, playing a fundamental role in promoting greater standardization in medication-related processes. This tool facilitates the implementation of effective preventive and corrective strategies, promoting a culture of patient safety and improving the quality of pharmacotherapeutic services in the hospital environments evaluated.

The consolidated results obtained through the application of the evaluation tool in the four Costa Rican hospitals reflect a high level of implementation of safe medication practices, while non-implemented processes represent only a minimal proportion. This level of implementation is comparable to the findings reported in Spain, where a multicenter study conducted in intensive care medicine services identified a notable level of compliance with these practices, with no relevant differences based on hospital size or type of service provided [16].

Similarly, in this study, notable differences were observed between the hospitals evaluated in Costa Rica, both in the overall percentage of implementation (sum of the results of each of the processes evaluated) (Table 1) and in the results of the implementation of specific processes (Figures 1-4). For example, while some hospitals achieved complete implementation in certain key processes, others demonstrated notably lower levels of implementation, or even none at all in some critical areas, as illustrated in the graphs in Figures 1-4.

Variability in implementation levels between hospitals is a common phenomenon, influenced by factors such as organizational culture, national and international standards applied, and the availability of specific resources in each institution [17].

These differences highlight the need to design specific strategies for each hospital, focusing on areas that require priority improvements. The application of tools such as the one used in this study facilitates not only the identification of these critical areas but also the standardization of safe practices in medication management. This homogenization is key to guaranteeing patient safety and raising the quality of the health system [18,19]. Likewise, the findings underscore the importance of aligning practices with international standards and strengthening institutional resources, essential elements to optimize safety in pharmacotherapeutic processes.

The analysis of medication errors reported between January 2020 and January 2024 by Hospital A, the study center, and the only one that provides these data, identified a total of 1,639 errors during this four-year period. These errors were distributed throughout the different stages of the medication process, with the prescription and transcription phases standing out as the most vulnerable, with 43.07% (n=706) and 30.14% (n=494) of errors, respectively.

The combination of data from the cross-sectional survey and retrospective analysis provided a comprehensive understanding of medication error management in private hospitals. While the survey offered a broad perspective on the implementation of good practices in four institutions, the retrospective analysis of Hospital A allowed for a detailed exploration of specific errors, their stages, severity, and types of drugs involved.

It is estimated that during the pandemic period, between 50% and 60% of medication errors occurred during the prescription stage [20,21]. Similarly, a study carried out in 2021 in a hospital in Indonesia allows these findings to be compared with international data, revealing that prescription errors were also the most frequent type of medication error, accounting for 54.40% of the total errors recorded [22]. Although the period of analysis of both studies does not coincide, these data provide a relevant perspective on the behavior of this type of error in different contexts, highlighting the importance of implementing global strategies to prevent them and improve patient safety.

These results reflect the high susceptibility of these stages due to the inherent complexity of the processes, the reliance on accuracy in communication, and potential limitations in support systems [23].

It is especially important to point out what a prescription error consists of since this stage is the starting point of pharmacological treatment and has a cascading effect on the rest of the process [24]. Prescription errors encompass critical activities such as labeling, packaging, dispensing, patient education, and monitoring during medication use. Identifying and addressing these issues is critical to ensuring patient safety, as any failure in this phase can lead to serious adverse consequences that affect the efficacy of treatment and put the patient's life at risk [25,26].

The analysis of medication errors occurring during the study period, classified according to their severity, showed that, as can be seen in Figure 5, most of the errors reported corresponded to Category B according to the NCC MERP classification. This implies that most errors were identified before reaching the patient, which reflects the effectiveness of the prevention and early detection systems implemented in the institution.

A notable agreement is observed with the results obtained in other studies reported in the literature. For example, in a study conducted in Mexico between June 2020 and September 2022, 37% of medication errors reported during that period were also classified as Category B, corresponding to the category with the most error reports [27]. This suggests that, regardless of the geographical context, health systems that implement effective monitoring strategies are able to detect a noteworthy proportion of errors in the early stages, thus avoiding negative consequences for patients [28]. These results reinforce the importance of strengthening prevention and screening mechanisms at all levels of the medication process as a key strategy to improve patient safety [29,30].

Likewise, the results show that the medication errors during the study period were predominantly associated with widely prescribed drugs, followed by high-risk drugs, including anticoagulants, opioids, antineoplastic agents, or concentrated electrolytes, and, to a lesser extent, vaccines. This behavior coincides with what has been observed in the results of other studies where the most common errors tend to involve drugs widely used in clinical practice, such as analgesics, anti-inflammatories, and laxatives, mainly due to their high frequency of prescription and administration [2,31].

It is estimated that widely prescribed drugs account for more than 50% of errors reported in medium- and high-complexity hospitals and approximately 38% in high-risk drugs [31]. 

In general, widely prescribed drugs tend to be more involved in prescription and transcription errors, while high-risk drugs excel in errors during administration, due to the requirement for greater precision in their handling [31-33].

While it is true that widely prescribed drugs have a lower potential for harm than high-risk drugs or vaccines, their high turnover in pharmacies increases the impact on patient safety. This underscores the need to implement prevention strategies targeting not only high-risk drugs but also those in widespread use, improving prescribing, reviewing, and administration processes. Communication strategies are of paramount importance in reducing errors, along with a multi-center approach that includes technology, pharmacist involvement, standardized processes, and strong safety culture that are essential for the mitigation of medication errors [34-35].

Limitations of the study

This study has several limitations that should be considered when interpreting its results. First, the retrospective analysis was based exclusively on Hospital A data, which restricts the generalization of the findings to other private hospitals in Costa Rica, especially those with limited resources or less developed practices in medication error management. In addition, the cross-sectional survey, although robust in its design, depended on the self-reporting of the participating hospitals, which may introduce biases related to the accuracy or completeness of the responses. Despite these limitations, the study provides a solid basis for future research and strategies to improve patient safety in the private hospital context.

Likewise, no direct comparison between the participating hospitals was conducted. This decision was made due to the inherent differences in hospital levels and certifications. These factors influence the implementation of medication error management practices, making direct comparisons between institutions with varying complexities and accreditations methodologically inappropriate.

## Conclusions

This study provides critical findings on the management of medication errors in private hospitals in Costa Rica, emphasizing the prescription and transcription stages as the most vulnerable and highest-priority areas for intervention. The effectiveness of existing error detection systems was also demonstrated, particularly in Hospital A, where a notable proportion of errors were intercepted before reaching patients. To address these issues, targeted training programs should focus on mitigating vulnerabilities in these critical stages, while advanced technologies like electronic prescribing and automated dispensing systems can further enhance safety. Standardized protocols must be reinforced to ensure consistency, and foster a culture of open, non-punitive error reporting is essential for continuous improvement. Expanding the use of the evaluation tools to public hospitals and other institutions in the region could help identify specific challenges and promote standardized practices. Aligning local procedures with international standards would strengthen healthcare systems, enhance patient safety, and improve treatment outcomes, offering a comprehensive approach to advancing medication error management and healthcare quality.

## Appendices

Appendix A 

Medication Error Management Process Assessment Tool

**Table 3:** Medication Error Management Process Assessment Tool *ME: medication error; AEs: adverse effects; NCC MERP: National Coordinating Council for Medication Error Reporting and Prevention

	Indicator			
	Not implemented	Partially implemented	Fully implemented	Feedback
1. Planning Safe Medication Practices				
A medication safety self-assessment protocol is in place				
There are strategies for reducing errors in high-risk populations, high-risk processes, high-risk drugs, and drugs that look and sound the same				
There are strategies that allow to prevent ME				
There are strategies that make ME visible				
There are strategies that mitigate damage if an ME occurs				
There are strategies for risk reduction that are based on the literature				
Strategies are available that reduce medication risks associated with drugs that look and sound the same				
2. Organization and Management				
A written document is told that addresses medication processes and identifies how medication use is organized, managed, and monitored throughout the hospital				
All environments, services, and individuals who manage medication processes are included in the organizational structure				
A licensed pharmacist directly oversees the pharmacy's activities or pharmacy services and ensures compliance with applicable laws and regulations				
At least one review of the medication management system is documented annually, covering medication processes				
There is a uniform system of dispensing and distributing medicines that guarantees the applicable laws and regulations				
Appropriate sources of medication information are readily available to those involved in medication use				
A antimicrobial stewardship is in place and implemented that involves doctors, nurses, and professional pharmacists in infection prevention and control				
There is a antimicrobial stewardship that is based on scientific evidence, accepted practice guidelines, and local laws and regulations				
There is a antimicrobial stewardship that has guidelines for the proper use of these drugs, including the proper use of prophylactic antimicrobial therapy				
There is a mechanism that allows the supervision of the antimicrobial stewardship				
Monitoring the effectiveness of the antimicrobial stewardship				
3. Selection and Acquisition of Medicines				
There is a list of medications by brand name or generic name, stored in the hospital or readily available from external sources				
The list of medications stored in the hospital or readily available from external sources is reviewed annually				
There is a process used to develop and monitor the medication list that includes representation of healthcare professionals involved in the process of ordering, dispensing, administering, and monitoring patients in the hospital				
There is a process for getting medications overnight or when the pharmacy is closed, and for getting medications that are not in "stock" or are not normally available at the hospital				
Formularies are designed to improve the safety of drug use and not just as a cost-saving measure				
The pharmacist considers whether a drug being reviewed for entry into the formulary has potential safety concerns				
Strategies to prevent ME are considered when entering medications with high potential errors				
The number of concentrations available is standardized and limited				
The "rule of 6" is not used				
Implementation of consistent terminology in cases requiring more than one concentration and consideration of the use of additional labeling to distinguish between different concentrations				
Continually updated with literature regarding drug safety warnings				
Ongoing review and analysis of reported ME data is conducted and should be handled by a safety committee or pharmacotherapy committee				
Implementation of a continuous mechanism to respond to updates on drug safety				
The pharmacy department is responsible for all procurement of medications within the hospital				
No medications are brought in from outside sources without prior consultation with the pharmacy department				
Medical samples are not used as inpatient treatment, but only as outpatient treatment with implementation of appropriate policies and procedures				
The patient's own medications are used only after the authorization of the doctor in charge of the hospitalization and the identification of the pharmacist				
Pharmacists are involved in evaluating purchasing decisions (what is purchased, how much is purchased, and when it is delivered) and replacement of all medication devices (inhalers) and are included in discussions related to devices that use medications for operational requirements (dialysis machines)				
Policies and procedures are in place to ensure the safety and continuity of care for patients who are admitted to the hospital with permanent elastomeric pumps				
Procedures are in place to communicate drug shortages to prescribers and clinical staff.				
When there is a shortage of medicines, alternatives and substitution protocols are provided to the hospital's clinical staff				
In cases of severe shortages of medicines, a contingency plan is in place in collaboration with the affected doctors and the health system committees				
Drug alternatives undergo a safety analysis before recommendation				
4. Storage				
Medicines stored in the pharmacy are under the right conditions to maintain the stability of the product, according to the manufacturer's instructions				
There is a protocol for storing medicines outside the pharmacy				
Controlled substances are handled in accordance with applicable laws and regulations				
Medicines or products that are high risk or require special handling are properly labelled, stored and monitored				
Medicines and raw materials are properly labelled with their contents, expiry dates and warnings				
Concentrated electrolytes found outside the pharmacy are only found in stop carts or properly insured sites				
All medication storage locations in the hospital have a loss or theft protection system				
The stop carts are in fixed and easily accessible places so that they allow the proper handling of emergencies				
A protocol for the storage of emergency medicines is in place and implemented, which includes their monitoring and maintenance				
The stop cart allows the protection and unlocking of medicines without the need for a key or specific person				
Emergency medications are monitored and replaced in a timely manner after use or in case of damage or expiration				
A risk-based antimicrobial stewardships is used for the identification and implementation of strategies to improve the efficiency and accuracy of medication delivery during emergency resuscitation				
There is a process for receiving recall notices when there is a problem during the manufacture or distribution of the drug				
A recall protocol is in place that includes the proper and safe identification, recall, and return or destruction of recalled drugs by the manufacturer, supplier, or regulatory agency				
The recall protocol includes medications formulated within the hospital, as well as the recall of products that have been used				
In all protocols for the use of medicines, a homogeneous nomenclature is used to avoid ambiguities				
In all hospital protocols, medicines are mentioned by active ingredient and not by brand name, except for combination products				
There is a protocol for the detection of expired medicines and products and a schedule is implemented to periodically inspect and withdraw expired medicines				
A protocol exists and is implemented for the handling or destruction of expired medicines				
An inventory management process is in place to reduce the risk of errors associated with drug shortages and expiration				
There is a rotating system and monitoring of cold chain medicines to detect expired medicines				
There is a quarantine area for the storage of expired medicines, to prevent them from being used				
There is a consumption report that allows the selection of medicines and the quantities of these that must be stored				
The drugs are packaged ready for use in single dose				
The purchase of medicines packaged in large quantities or multi-dose vials is avoided, to avoid waste				
Medications that require extensive dilutions or calculations are not stored, if these medications must be stored, barcodes are used				
Medications are kept in storage until the time of administration, and any unadministered doses are returned to a controlled storage location				
Nurses only return leftover medications to the automated dispensing cabinet return container				
Systematic inventory audits are performed to identify and remove expired and low-use products				
5. Patient Intake				
A medical history is obtained, and a medication reconciliation is performed and reviewed within 24 hours of the patient's admission				
6. Medical Orders/Orders, Transcription and Review				
There is an orderly and easy-to-consult method with information about the patient's medications, both those that were reconciled and those that were prescribed during their current hospitalization				
The patient's medical records contain a list of the reconciled medications that the patient used before being admitted to the hospital				
Initial medical prescriptions include the list of medications that were used by the patient prior to admission, according to the process established at the hospital				
Only personnel authorized by the hospital and by the relevant licenses, laws, and regulations may prescribe and execute medical orders				
A process is in place to set limits on prescribing practices and place medical orders				
The personnel authorized to prescribe medicines is known to the pharmaceutical service in charge of dispensing medicines				
A protocol is established and implemented for the training of personnel on the process for prescribing medicines, as well as for the safe transcription of medical indications				
All prescriptions and prescriptions contain the name, dosage, frequency, and route of administration of the medications				
For the dispensing of medicines in the pharmacy, it is required that all medical prescriptions contain the following elements, without exception: full name of the prescriber, address, telephone number, name of the patient with both surnames, age and identification number, the name of the medicine or medicines, pharmaceutical form, potency, quantity, route of administration and dosage, signature of the prescriber and professional code number, and date of prescription of the prescription				
A process is in place to manage the handling of medical prescriptions that are incomplete, illegible or unclear, and measures are included to prevent this from happening further				
There is a system of stopping or "hitchhiking" prescriptions				
Medications that were prescribed during hospitalization are inserted into the patient's medical record at the time of discharge or transfer				
Dispensing medications for intravenous administration requires that the prescription specify the strength of the medication, the speed and time of administration				
An electronic prescription record is available, and if not available, written medical orders are legible				
Unclear medical orders are reported as ME				
Verbal or telephone medical indications are reserved only for situations in which it is impossible for the physician to write or enter the order into the electronic system				
The use of prescriptions for the suspension of medications is avoided. If necessary, the instructions are clear and include the duration of the discontinuation or a clearly identified time for continuation, otherwise the medication is renewed/reordered				
Active systems or reminders are established that notify the prescriber that therapy is to be discontinued for safety reasons				
Automatic dosing protocols: substitutions of therapeutic classes, change of route of administration, dose adjustment due to deterioration in renal or hepatic function, rounding of doses and discontinuation orders, are clearly written and included in the patient's record				
The medical order must be clear before the prescriber leaves the patient's care unit; In case the doctor has left the unit, the nursing staff must contact him or her before transcribing the order				
The pharmacy does not process incomplete medication orders				
A system is in place to minimize the use of error-prone abbreviations				
In dose rounding, a leading zero should always be used before placing a decimal and a trailing zero after the decimal is never used				
The transcription process is completed in a quiet, well-lit area away from distractions				
A system is in place to compare the medication administration record with active orders				
There is a system for verifying the transcription of medical indications				
The review of the medical prescription is carried out before the preparation and dispensing of the medicines				
7. Preparation and Dispensing				
The preparation and dispensing of medicines comply with laws, regulations and standards of professional practice				
Medications are prepared and dispensed in clean, clear, safe, and functionally separated areas with appropriate medical equipment and supplies				
Personnel who formulate or prepare sterile products or medications are trained and competent in medication preparation principles and aseptic techniques and are provided with resources to support the medication preparation process				
A guideline for the use of single-use and multi-dose vials in medication processes exists and is implemented				
Medications stored, prepared, and dispensed from areas outside the pharmacy comply with the same cleaning measures required in the pharmacy				
The specific information required from the patient for a prescription review process prior to dispensing is defined, and the information is always available				
Each prescription or medical order is reviewed to determine its suitability according to the elements required by current regulations				
Individuals who are permitted to conduct prescription review are deemed competent to do so, and are provided with resources to support the review process				
If the designated licensed practitioner is not available to perform a full prescription review, a trained person perform and documents a full review within 24 hours of dispatch of the following critical items: drug adequacy, dosage, frequency and route of administration, therapeutic duplication, allergies, drug interactions, patient weight, Contraindications, toxicity				
The review is facilitated by the availability of appropriate clinical information for all patients receiving medications, and this clinical information is always available in the pharmacy				
The programs and support materials used for the complete review of the prescriptions are updated				
Medications are dispensed in the most ready-to-administer form available				
The system supports accurate and timely dosing and documentation of dosing practices				
After preparation, medications that are not administered immediately are labeled with the medication name, dosage or strength, preparation date, expiration date, and two patient identifiers				
The preparation of medicines is carried out under adequate conditions of sanitation, temperature, light, humidity, ventilation, segregation and safety, to guarantee the integrity of the medicines and the safety of health personnel				
When a pharmacy technician prepares a drug or product for dispensing, a pharmacist must always perform a double check				
When the preparation of medications or products is carried out outside the pharmacy area, the department implements policies and procedures for the verification of these				
Double verification includes checking the components used, their quantities and their expiration dates				
The medicines are available for hospital use in unit containers for use and ready to be administered and without the need for additional handling by the personnel administering the medicine				
It prevents the personnel administering the medicine from having to remove doses from the containers, reconstitute powdered pharmaceutical products, split tablets, etc.				
Oral products that are not available in single-use containers are repackaged				
The staff is adequately trained for the preparation of sterile and non-sterile products; facilities comply with standards and regulations; staff maintain and document all processes and procedures for which they are responsible				
Documents are available indicating storage requirements, expiry dates of products and dates of subsequent use of preparations to ensure product integrity				
Aseptic techniques are implemented in all sterile product preparations and the staff is adequately trained and regularly audited to determine their competence				
A labelling system is in place to clearly identify the sterile product that was prepared, the concentration, its stability and expiry date				
Any medication that is dispensed in non-emergency situations is pre-checked by a pharmacist				
All medications that are dispensed or dispensed by the pharmacist are checked the original medical prescription				
The pharmacist ensures that all work done by support staff or using automated devices has been done correctly				
The pharmacist participates in self-verification processes by reading prescriptions, labeling, and dose calculations				
A second pharmacist verifies the operation of high-risk pharmacological products (chemotherapy, pediatric drugs, total parenteral nutrition)				
The pharmacist makes sure that the drug, labeling, packaging, quantity, dosage, and instructions of the high-risk drugs are correct, and a second check is done, by a second pharmacist				
There is a list of medications with the corresponding indications in cases in which there may be a prescription rejection				
8. Administration				
Proper identification of personnel authorized to administer medications is in place				
There is a record of the administration of each dose of medication				
Verification of medications, dosage amount, and route of administration with prescription or doctor's order				
Patients are informed about the medications to be administered and can ask questions				
Medications are administered as prescribed, in a timely manner, and noted in the patient's medical record				
A risk assessment is done for reconciled medications that addresses where and when medications were obtained and how they were stored in the home				
A process is in place to regulate the patient's self-administration of reconciled medications				
A process exists and is implemented to govern the handling, use, and documentation of medications that were reconciled at the time of hospital admission				
A process is in place that specifies the storage, monitoring, and use of medication samples within the hospital				
A risk assessment system is in place for medical samples that were reconciled at the time of hospital admission				
A process is in place to regulate the availability, management, use, and documentation of drug samples				
Double checks are performed prior to administration of high-risk medications, including performing calculations and checking for the patient's allergies				
A process is in place for the verification of two patient identifiers prior to medication administration				
Personnel authorized to administer medications are properly trained in administration procedures				
Effective and recurrent medication management education programs are established				
Staff education and training documentation systems are in place and training is conducted on a regular basis				
9. Monitoring				
Monitoring and documentation of drug AEs in patients is carried out when appropriate				
Drug AEs in patients are monitored according to defined protocols				
There is a standardized process for recording drug-related AEs in the patient's medical record				
A standardized process is in place for reporting drug AEs as part of the hospital's quality program				
AEs are reported within the time frame established in the protocol				
There is a definition of ME and possible ME, and there is also a classification according to the NCC MERP criteria				
A protocol for the reporting and resolution of ME and potential ME is established and implemented				
Staff are established to carry out ME reports to the hospital quality department				
Information about ME and potential ME is used to improve processes related to drug use				
There are guidelines for the adequate and correct collection of blood samples for the determination of serum drug concentrations and laboratory values necessary for the monitoring of drugs commonly associated with ME				
Clear and available documentation of the values that are monitored with specific normal ranges is available to facilitate the interpretation of results				
The staff is trained to identify the most common AEs that patients present after the administration of a medication and a process is in place for the management of these				
Hospital staff are trained to monitor the effectiveness of medications				
The staff is trained to optimize the patient's pharmacotherapy and there is a protocol to follow in cases of an inadequate response to the medication				
Hospital staff involved in prescribing, dispensing, and administering medications are trained to interpret patient data				
Flow charts are available that standardize medication administration times and patient monitoring times after administration				
The monitoring time of titratable drugs (heparin, vasopressors, insulin) is included in the set of medical indications				
There is a record of the values with date and time, which allows the creation of an appropriate frame of reference				
10. Patient discharged				
Pharmacists participate in multidisciplinary discharge committees and are involved in the medication reconciliation process before the patient is discharged				
The patient is provided with an accurate list of medications to take after discharge				
11. Evaluation				
Continuous evaluation of ME prevention systems and processes is maintained				
